# Introduction of a breast cancer care programme including ultra short hospital stay in 4 early adopter centres: framework for an implementation study

**DOI:** 10.1186/1471-2407-7-117

**Published:** 2007-07-02

**Authors:** Mascha de Kok, Caroline NA Frotscher, Trudy van der Weijden, Alfons GH Kessels, Carmen D Dirksen, Cornelis JH van de Velde, Jan A Roukema, Antoine VRJ Bell, Fred W van der Ent, Maarten F von Meyenfeldt

**Affiliations:** 1 Department of Surgery, University Hospital Maastricht, Maastricht, the Netherlands; 2 Department of Radiology, University Hospital Maastricht, Maastricht, the Netherlands; 3 Department of General Practice/Centre for Quality of Care Research/Care and Public Health Research Institute (CAPHRI), Maastricht University, Maastricht, the Netherlands; 4 Department of Clinical Epidemiology and Medical Technology Assessment (KEMTA), University Hospital Maastricht, Maastricht, the Netherlands; 5 Department of Surgery, Leiden University Medical Center, Leiden, the Netherlands; 6 Breast Unit, St. Elisabeth Hospital, Tilburg, the Netherlands; 7 Department of Surgery, Laurentius Hospital, Roermond, the Netherlands; 8 Department of Surgery, Orbis Medical Center, Sittard, the Netherlands

## Abstract

**Background:**

Whereas ultra-short stay (day care or 24 hour hospitalisation) following breast cancer surgery was introduced in the US and Canada in the 1990s, it is not yet common practice in Europe. This paper describes the design of the MaDO study, which involves the implementation of ultra short stay admission for patients after breast cancer surgery, and evaluates whether the targets of the implementation strategy are reached. The ultra short stay programme and the applied implementation strategy will be evaluated from the economic perspective.

**Methods/design:**

The MaDO study is a pre-post-controlled multi-centre study, that is performed in four hospitals in the Netherlands. It includes a pre and post measuring period of six months each with six months of implementation in between in at least 40 patients per hospital per measurement period.

Primary outcome measure is the percentage of patients treated in ultra short stay. Secondary endpoints are the percentage of patients treated according to protocol, degree of involvement of home care nursing, quality of care from the patient's perspective, cost-effectiveness of the ultra short stay programme and cost-effectiveness of the implementation strategy. Quality of care will be measured by the QUOTE-breast cancer instrument, cost-effectiveness of the ultra short stay programme will be measured by means of the EuroQol (administered at four time-points) and a cost book for patients. Cost-effectiveness analysis will be performed from a societal perspective. Cost-effectiveness of the implementation strategy will be measured by determination of the costs of implementation activities.

**Discussion:**

This study will reveal barriers and facilitators for implementation of the ultra short stay programme. Moreover, the results of the study will provide information about the cost-effectiveness of the ultra short stay programme and the implementation strategy.

**Trial registration:**

Current Controlled Trials ISRCTN77253391.

## Background

Opinions on more optimal working methods ('best practice') for breast cancer treatment have been changing continuously[1-6]. Increasingly, implementation studies are applied investigating techniques how to change clinical practice to integrate 'best practice' recommendations[7,8]. Whether a new programme is consolidated after implementation depends on the quality of care, the cost-effectiveness of the innovation as such, and of the cost-effectiveness of the innovation incorporating the costs of implementation[9].

Breast cancer causes a prominent burden on the health care budget. Of the total costs of breast cancer treatment 35–50%[10] is spent on surgical treatment, of which the largest part is on account of the hospital stay. Since the 1990s, hospital stay has decreased from 10–14 days to 5–7 days and further to the level of day care admission[11-15]. Three randomised clinical trials on the subject of early discharge (2–4 days vs. 5–10 days) reported no adverse effects and lower illness rates, high patient satisfaction and good clinical outcomes for the early discharge as compared to the standard admission group[16-18].

A decrease in admission time should not affect quality of care in a negative way. Therefore, it is important to introduce a structured programme such as has been developed at University Hospital Maastricht (uhM) that includes current quality of care criteria[4,5,19]. Moreover, criteria for education, support and evaluation of the breast cancer care programme can (provide information to) enhance quality of care. Measurement of quality of care is of fundamental importance when evaluating a new programme.

At the uhM, an ultra short stay programme for breast cancer surgery was developed and evaluated, incorporating the aspects mentioned above. The programme consists of a structured care organisation including education and counselling, dedicated anaesthesia, active participation of the patient in her own treatment plan and in the decision to go home, and home care nursing. NABON guideline quality indicators are also incorporated in the programme (e.g. access time for the first visit to the Breast Unit, time spent in the process, number of breast conserving procedures and all diagnostic and treatment decisions being made in an appropriate multidisciplinary setting)[19]. Although the uhM programme was successful in the end, the programme was greeted with scepticism by both health care providers and members of the local breast cancer patient association. For example, in the beginning patients were discouraged by ward nurses to go home in the evening following surgery for the nurses did not trust the quality of the programme at that moment. In addition, it suffered from problems at the anaesthesiology department (e.g. administration of opioids at the recovery unit postoperatively even if patients did not suffer pain) and from organisational flaws. For instance, the day care surgery unit closed at 1800 hours and, therefore, patients operated on late in the afternoon had less recovery time as compared to those operated on early in the morning. Following on those limited opening hours, patients in need of more time to recover after 1800 hours were transferred to the inpatient ward from which discharge the same evening was rarely performed.

Traditionally, implementation strategies have been chosen for pragmatic reasons guided by personal acquaintance with a particular strategy[20]. In this project we chose to perform an extensive diagnostic analysis[20] of the problem, consisting of three issues:

1) how is current care organised?

2) how does it deviate from optimal care according to the innovative care programme?

3) what are barriers and facilitators for implementation of the ultra short stay programme?

The uhM experience accumulated valuable insight and knowledge about implementation of an ultra short stay programme for breast cancer. If such a programme is implemented on a wider scale, in other hospitals, these experiences may contribute to the design of implementation strategies that are tailored to local needs.

The implementation strategy will be developed on the basis of the results of the diagnostic problem analyses, and tailored to the needs of the stakeholder groups. The insights generated from this study may be translated into generally applicable insights on implementation of the innovative care programme on a larger scale while again defining patient benefits and demonstrating cost containment through the type of care provided.

### Aim and objectives

#### Aim

The aim of this study is to implement a programme incorporating breast cancer surgery in an ambulatory/24 hour stay setting, (tailored to the needs of these hospitals).

#### Objectives

To evaluate:

1) whether the percentage of patients treated in ultra short stay increases without an increase in the number of complications.

2) whether the quality of care as perceived by patients between the baseline measurement and measurement after implementation does not deteriorate.

3) the cost-effectiveness of the ultra short stay programme versus care as usual (programme cost-effectiveness) in the baseline period.

4) the cost-effectiveness of implementation of the ultra short stay programme versus 'doing nothing' (policy cost-effectiveness).

5) the actual performance of the professionals in the test hospitals according to performance indicators.

6) the perceived barriers and facilitators for the implementation of the programme incorporating surgery in ambulatory/24 hour stay setting in the participating hospitals so others preparing to change to ultra short stay care can profit from this information.

## Methods/Design

### Study design

The MaDO study is a multi-centre pre-post uncontrolled prospective study and involves breast cancer patients who are operated on with curative intent. Both pre and post- measurements last six months with an implementation period of six months in between.

### Selection of hospitals

The study is performed in four hospitals which have been selected on three criteria. First, all participating hospitals had to be recognised as early adopters (an active group with high status within the target group)[21] within the field of breast cancer treatment, expressed by the willingness to appoint a full-time breast nurse. Second, we wished to include one university hospital, one large training hospital, one small training hospital and one non-training hospital to cover the main organisational hospital settings in the Netherlands, and to assess whether difference in results could be explained by differences in setting. Third, recruitment of people from different hospitals from different parts in the Netherlands, enables us to have a representative sample of breast cancer patients in the Netherlands

### Selection of patients

#### Inclusion criteria

All consecutive patients from the four participating hospitals, aged over 17 years, diagnosed with breast cancer, and scheduled to undergo surgery are eligible for participation in the study.

#### Exclusion criteria

Concerning surgical techniques, there is no contra-indication for ultra-short stay. Patients whose physiology impedes participation, as assessed by the breast surgeon and breast nurse, are excluded from the study. Patients who cannot rely on sufficient informal care in the home situation during the first night following surgery and patients with complaints that necessitate postoperative monitoring (e.g. cardial, pulmonal or neurological diseases) are scheduled for at least one overnight stay. This decision is made in consultation with the anaesthesiologist. The breast nurse scores the reasons for patients who do not participate.

### Informed consent

Eligible patients are informed about the study by the breast nurse during a consultation separate from the consultation in which they are informed about the diagnosis breast cancer. Patients are given an informed consent form by the breast nurse when they are informed about the study, and they are asked to return the signed informed consent within a week. The informed consent regards consent to being asked to complete questionnaires with the aim to evaluate the cost-effectiveness of (the implementation of) the innovative care programme.

### Sample size calculations

In the uhM the percentage of patients discharged within 24 hours has increased from 13% to 84%. Our assumption is that the clinically significant difference of ultra short stay admissions between the pre and post measurement is at least 30% in all participating hospitals. To achieve this percentage, 40 patients are sufficient for both the pre and the post measurement in each of the hospitals to demonstrate statistical relevance (p < 0.05) with a power of 0.90[22].

### Intervention: the breast cancer care programme including ultra short stay

The intervention in this study concerns the comprehensive care programme for breast cancer surgery in ultra short stay (= ambulatory/24 hours stay setting). The key figures in the programme are the multidisciplinary team, the breast nurse, and the patient.

Traditionally, the surgeon has been the specialist who analyses the type of breast pathology presented. Preventive procedures, early diagnosis of non-palpable lesions, breast conserving therapy, and lymph node sparing therapy have raised possibilities to reduce the burden of breast (cancer) surgery. The role of the breast nurse was introduced to improve patient counselling. Through these developments, diagnosis and treatment of breast pathology have gained a more multidisciplinary character. A decrease in the burden of surgery may limit the need for hospital-based supportive care. Similarly, this leads to a demand for strict coordination of the different steps and disciplines involved. Moreover, responsibilities for aspects of care need to be reallocated to other persons: from hospital- based supportive caregiver towards informal caregiver, from clinician to nurse specialist, from in house nursing staff to outpatient nursing staff, from hospital-based nursing staff to home care nursing staff etc.

While the clinician's activities are limited to solving medical problems, the breast nurse performs all coordinating tasks to create a programme that runs smoothly.

The patient is the key figure in the process. In a one-hour consultation, following the preoperative diagnosis, the patient is informed by the breast nurse about all aspects of surgery (preparation, day of surgery, medication etc.). A checklist is used for this consultation and all oral information is supported by written information. Parts of this information (e.g. information on home care nursing and what to do in case of problems) are repeated on the morning of surgery, and at the moment of discharge.

### Implementation strategy

The first step in the implementation process concerns the assessment of the guideline recommendations by consensus among experts, who define the complete set of recommendations (n = 29) as well as a set of key recommendations (Table 1).

**Table 1:** Key recommendations of the study guideline to enable ultra short stay for breast cancer surgery. The complete guideline contains 29 recommendations. The participating breast nurses and surgeons from the study groups rated all recommendations and decided upon which recommendations would be defined as 'key' recommendations.

• The treatment of all breast cancer patients is planned in a weekly multidisciplinary meeting.
• The interval between referral and first visit to the breast unit is 5 working days or less.
• The interval between diagnostic tests and informing patients about their results is 5 working days or less.
• The interval between the decision to operate and surgery is 15 working days or less.
• The number of preoperative hospital visits is minimised.
• The general practitioner is informed about diagnosis, treatment plan and potential side-effects prior to surgery.
• Patients planned for day care treatment are postoperatively given the choice between continuation of admission and discharge.
• Information given to patients about discharge is supported by written information.
• Decisions on patient discharge are based on clear guidelines.
• Specialised home care* for patients in the period following surgery is facilitated.
• The breast nurse stays in contact with the patient during the postoperative period.
• At least one night of hospital admission is planned for patients
with co-morbidity and/or insufficient postoperative supervision.
• The breast nurse informs the patient about the need for informal care in the home situation.

A so-called diagnostic analysis assesses the usual care, and explores factors that impede or facilitate the bridging of the gap between usual care and the programme to be implemented. Information for the diagnostics analysis will be retrieved from interviews during outreach visits, meetings, and telephone and e-mail conferences. The results of the assessment of usual care in the participating hospitals are compared to the standards of the innovative care programme. Information on perceived barriers and facilitators for implementation of the ultra short stay programme will be collected during outreach visits to the hospitals. These visits will be conducted in the recruitment and preparation phase. They will be aimed at preparation of the actual implementation and listing of the barriers and facilitators that were perceived by the participants for implementation of the care programme. This set of barriers and facilitators will be described on different levels: study guideline, care provider, patient, colleague, organisation, and financial resources and reimbursement[23].

Subsequently, a hospital-specific strategy is applied to implement a hospital-tailored version of the comprehensive care programme. This strategy is aimed at overcoming the barriers. Multi-faceted implementation strategies are used, providing insight, inducing change and acceptance, and feedback to maintain changes. These strategies are based on several components: 1) The promise that each early adopter makes to the project leader (MvM) to appoint a breast nurse fully available for coordination for the programme; 2) High-frequency outreach visits and study group meetings representing all disciplines involved in breast cancer care, provide the forum in which all steps for implementation are prepared, and issues are discussed and resolved. This local working party is also responsible for the communication of progress of implementation and of the findings within the hospital, to patients, and to other involved parties.; 3) The care process is measured through indicators mentioned in the Case Record Forms that are scored within the hospital, followed by feedback on performance by the researchers.

### Main indicators (Table 2)

**Table 2:** Main indicators of the MaDO study.

*Outcome indicators*
• Final type of admission
• Readmission rate
• Complication rate
• Quality of care from the patients' perspective
• Cost-effectiveness of the ultra short stay programme
• Cost-effectiveness of the implementation strategy

*Process indicators*

• Involvement of home care nursing
• Performance of professionals according to the protocol
• Reasons why patients were not treated according to ultra short stay protocol

#### Measurements and timing of measurements (Figure 1)

**Figure 1:**
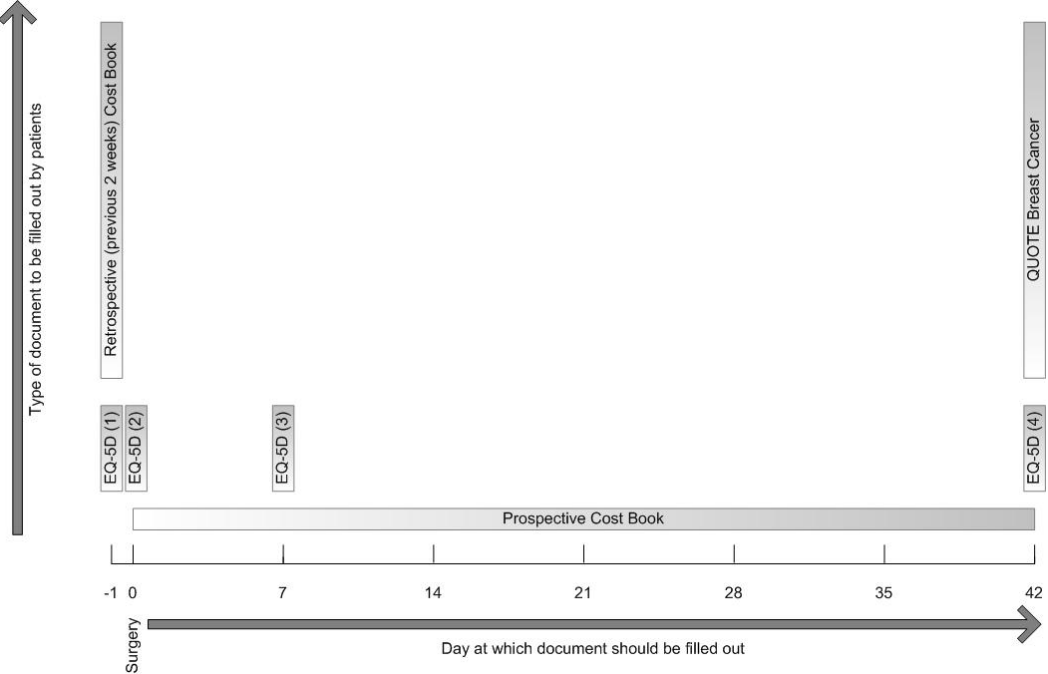
**Overview patient questionnaires**. Overview of the type of questionnaires that are offered to patients and the time at which these questionnaires should be filled out.

- Information on the achievement of the targets of the implementation strategy is collected through Case Record Forms.

- Quality of care is measured from the patient's perspective through the QUOTE-breast cancer, a questionnaire that was developed by and for breast cancer patients and concerns those aspects that breast cancer patients perceive as most important when assessing quality of care[24,25]. The QUOTE-breast cancer questionnaire is filled out six weeks after surgery.

- Information on the performance of professionals is gained through Case Record Forms. The performance indicators have been developed by means of a consensus procedure in the uhM project group.

- Information on societal costs are retrieved from hospital information systems and through a prospective cost book that is filled out by patients during six weeks from the moment of discharge. The day before surgery, patients are asked to fill out a retrospective cost book to assess the patients' health-care related costs for a period of two weeks prior to surgery.

- Patients fill out EuroQol questionnaires at baseline (one day before surgery) and at 24 hours, one week and six weeks after surgery.

When patients sign informed consent and receive the questionnaires, a stamped envelope is given to them which can be used to return the set of questionnaires.

Programme cost-effectiveness (as compared to care as usual) consists of the two components costs and effectiveness, and is expressed as incremental costs per Quality Adjusted Life Year (QALY) of the ultra short stay programme versus care as usual. Incremental costs are expressed as the difference in costs between the ultra short stay programme and care as usual. Costs are calculated by multiplying the volumes with the appropriate cost prices. Information on (societal) costs are retrieved from hospital information systems and through a prospective cost book that is filled out by patients during six weeks from the moment of discharge. Effectiveness of the programme is determined through the measurement of safety, (number of complications, number of visits to the emergency unit, number of re-admissions etc.; information gained from the Case Record Forms) and generic health-related Quality of Life. Generic health-related Quality of Life is recorded through the EuroQol-5D (EQ-5D)[26], which is completed at baseline, 24-hours after surgery, 1 week after surgery and 6 weeks after surgery. QALYs will be determined in two ways; from a societal perspective using the Dolan score, and from an individual perspective using the VAS score of the EuroQol. The multiple imputation method will be used for missing data analysis[27].

- Policy cost-effectiveness of implementation of the ultra short stay programme versus 'doing nothing' is expressed as incremental costs per Quality Adjusted Life Year (QALY). Costs of the implementation strategy is measured through cost forms that participants in the study groups fill out, through scoring by a researcher (MdK) of all other costs (e.g. times, materials, travel costs) in a data sheet. Effectiveness of the implementation strategy is measured through Case Record Forms containing treatment information. More details on programme cost-effectiveness and policy cost-effectiveness can be found in the section 'Economic evaluation'.

### Time schedule for the study

The study lasts three years. The first year of the study will be spent on the analysis of the breast cancer care processes in the different hospitals and the development of the measurement instruments. The following six months are used for baseline measurements, and preparation of the implementation of the new programme by the members of the local study groups. Recruitment of patients will be performed by breast nurses and advanced practice nurses working at the breast units. Then, six months are used for implementation and running of the actual programme, followed by six months of measurements 'after implementation'.

### Statistical analyses

Intention to treat will be used for data analyses, and analyses will be performed using the SPSS package, version 12.0.1 ^® ^for Windows (SPSS INC 1989–2003). P values < 0.05 will be defined as statistically significant. The pre and post comparison of the interval scaled outcome variables will be analysed with a multivariate linear regression model. Apart from the independent variable hospital, other independent variables included in this model are type of surgery, age, informal care at home, involvement of home care nursing and starting time of surgery. Furthermore, correlation of data within a hospital are accounted for by clustered data analysis.

### Economic evaluation

In the economic evaluation, programme cost-effectiveness will be combined with cost-effectiveness of the implementation strategy, to determine policy cost-effectiveness.

### Programme cost-effectiveness

The time horizon of six weeks seems appropriate as patients may receive additional treatment after surgery (e.g. radiotherapy, chemotherapy or hormone replacement therapy). Therefore, we expect that only short-term quality-of-life effects and costs can be attributed to the ultra short stay programme. Cost analysis includes health care costs and costs outside health care such as out-of-pocket costs or productivity costs due to reduction in paid work and/or domestic activities. Health care costs refer to hospital and other health care facilities costs. Hospital costs consist of personnel, material, capacity costs, and overhead, associated with the diagnosis, surgical treatment, hospital stay and follow-up. Other healthcare costs include costs of visits to the general practitioner, medication, home care etc. Health care costs are estimated by multiplying the resource utilization with the cost price per unit. Most of these costs are estimated by using existing resource registration systems and available cost prices through the financial departments of the participating centres. If true cost prices are not available in the participating centres, 1) existing cost prices of the uhM or 2) official directive prices are used[28]. Healthcare costs incurred outside the hospital and costs outside health care are partly estimated through a structured cost book that is kept by all participating breast cancer patients during the period of six weeks following discharge. Productivity losses due to absence from work are estimated through the friction cost method[28,29]. To perform the cost-effectiveness analysis, costs and effects of patients treated according to care as usual (before implementation) will be compared to costs and effects of patients treated according to the ultra short stay programme (after implementation).

### Cost-effectiveness of implementation

In each centre, the costs of the implementation process are calculated based on time and materials invested during the different phases of implementation. The diagnostic analysis may be looked upon as intervention. Its costs should be included in the costs of implementation. To determine cost-effectiveness of implementation, the costs of implementation will be weighed against the proportion of patients that is treated according to the ultra short stay programme.

### Policy cost-effectiveness

The cost-effectiveness of implementation, i.e. the extra cost per patient treated according to the ultra short stay programme, will be combined with the cost-effectiveness of the breast cancer care programme to determine policy cost-effectiveness, using Mason's model[9,30]. In this model, the duration of the effects of implementation is crucial for the cost-effectiveness of implementation, which will be investigated explicitly in this study. The incremental cost-effectiveness ratio of implementation of the ultra short stay programme versus 'doing nothing' will be expressed as the incremental cost per QALY.

## Discussion

Breast cancer surgery on an inpatient basis is a burden on the health care budget. Although previous studies have shown that ambulatory surgery is feasible for breast cancer patients, this has not been widely recognised in Europe. This is, to our knowledge, the first study that systematically evaluates the impact of an implementation strategy on patient and professional outcomes, patient experiences, costs, barriers and facilitators for change, for ultra short stay after breast cancer surgery.

A randomised controlled trial is usually preferred over any other design. However, for several reasons a randomised controlled trial was not regarded as feasible. The first reason concerns the expected lack of willingness on the part of hospitals to be allocated to the control condition as they would get nothing in return for their efforts had we chosen a design with a concurrent control group. The number of participating hospitals in the study is too small to allow a controlled study design. A controlled study would have led to an unacceptable rise in study costs and the study period was too short to construct a waiting list control group. However, we think that the numbers of patients from the four participating hospitals provide enough power to draw conclusions on the estimation of change.

Random allocation at patient level was not considered for legal and organisational reasons; patients always have the right to go home whenever they want, making randomisation for early or late discharge rather meaningless. Moreover, it is unethical to have one patient discharged the day of surgery while her neighbour is allowed to stay for another day or two. The extent of organisational changes to implement short stay within the existing processes of care are of such a scale that it is hardly feasible nor acceptable to allow different admission periods at the same time within one hospital.

We are aware that the decision for a pre-post design instead of a randomised controlled trial cannot rule out the influence of developments and ongoing changes in health care, including reductions in lengths of hospital stays. This aspect will, therefore, be taken into account when conclusions are drawn based on the results of the study.

In line with the most recent insights in implementation science a diagnostic analysis will be performed with the intention to tailor the implementation strategies to the needs of each hospital. However, this analysis will encompass a lot of time, energy, and costs, while there is no evidence as yet that such an analysis will assure a positive effect of implementation.

Since we wittingly selected merely early adopters, the external validity may be limited with regard to the smoothness of implementation. However, we think that although not every hospital can be labelled an 'early adopter', every hospital willing to participate in an implementation study must be eager to make the study a success, regardless of the degree of novelty at that time.

Although diagnostic analysis explores the willingness to implement as well as the opportunities for implementation, the number of perceived barriers and facilitators will be underestimated, or at least not overestimated. A thorough exploration of actual barriers and facilitators must, therefore, be performed during the actual implementation process.

There is a risk of contamination of the baseline measurement by socially desirable behaviour because the outreach visits for preparation of the study have taken place before and during the baseline measurement period. However, fixed moments to actually start with the innovative programme, are scheduled within each hospital and agreed upon. Feedback is given by the researchers if they suspect that hospitals may start the programme earlier than planned. Therefore, we think this influence is limited.

A cost-effectiveness analysis is performed on the ultra short stay programme itself, and on the implementation strategy used. The goal of this study is to implement the ultra short stay programme for breast cancer surgery aiming at improved patient information, improved organization of care, a high degree of quality of care, and a reduction in mean hospital stay which results in a reduction of health care costs.

## Abbreviations

MaDO- Breast cancer surgery in day care

QALY- Quality Adjusted Life Year

QUOTE- breast cancer – questionnaire on quality of breast cancer care (acronym for "QUality Of care Through the Eyes of patients"), developed through qualitative and quantitative methods with breast cancer patients

UhM- University Hospital Maastricht

## Competing interests

The author(s) declare that they have no competing interests.

## Authors' contributions

MdK is responsible for data collection and drafting the manuscript. MdK works under direct supervision of MvM and TvdW. CF, TvdW, CD, FK, CvdV, JR, AB, FvdE and MvM all participated in discussing the design of the study and developing the research protocol. They were co-applicants on the successful funding proposal. All authors read and corrected draft versions of the manuscript.

## Pre-publication history

The pre-publication history for this paper can be accessed here:


